# The Microscope in Sterility

**Published:** 1869-07-01

**Authors:** J. Marion Sims


### MISCELLANY.

### *The Microscope in Sterility.*

[Extracts from a paper read at the Oxford meeting of the British Medical Association in August, 1868, and subsequently read before the New York Medical Society on the 7th of December, 1868. Printed first in British Medical Journal in November, and reprinted in New York Medical Journal in January, and made into a pamphlet from this reprint.—*Nashville Journal.*]

BY J. MARION SIMS, M.D.

How often have we heard it said—how often have I said it myself—"An operation is necessary in this case, because the canal of the cervix is too small to permit the easy entrance of the semen!" Now, these important questions cannot be determined with any degree of accuracy in this hap-hazard manner. For it is not always necessary to incise the cervix uteri, simply because it does not easily admit the passage of an ordinary sound; nor, on the other hand, are we justified in condemning an operation, simply because the sound can be passed easily. In other words, a very small os does not call for operation, nor does a larger one always forbid it. Do you not think, then, that a great service would be rendered, if we could deduce this question of operation, or no operation, from the broad domain of speculative opinion to the narrow path of absolute scientific certainty? There is nothing easier, for the microscope accomplishes this in the most perfect manner imaginable. It settles the question of operation, or no operation, in an instant, leaving nothing whatever to be guessed at and nothing to be desired.

Is it surprising that positive knowledge of this sort should meet with opposition among honest, earnest cultivators of medicine? Not at all. For it is ever so with any great truth. It must be first opposed, then ridiculed, after a while accepted, and then comes the time to prove that it is not new, and that the credit belongs to some one else. The truth here announced has had its day of opposition, and it must now soon take its stand as established and acknowledged.

On the subject of the microscopic examination of the utero-vaginal secretions, I have been misrepresented, maligned, and positively abused by a few, both abroad and at home; and I have been misunderstood by many who have not taken the trouble to read, to investigate, to think and to reason for themselves. And, Mr. President, under these circumstances, I cannot thank you too much for the high privilege of appearing here to explain and to defend my position by laying facts in the case before this learned Society, this great gathering of my countrymen, whose decision, I am sure, will be in accordance with truth and justice.

We may all differ honestly about abstractions, and theories, and mere opinions; but when it comes to facts and figures, there cannot long be a great difference among men of good common sense, with honesty of purpose in pursuit of truth. I have never yet been afraid of truth, however much it may conflict with prejudices, find it where I may; nor do I ever expect to see the day that I would fear to publish my convictions on any matter of professional importance, be the character of the opposition what it may; and, particularly, when I feel that these convictions are based upon facts that are immutable, and lead to results of the gravest importance to the honor of medicine, and to the advancement of knowledge. Whatever gives to any department of medicine greater exactitude, helps to raise it to the dignity of a science. And this is what I claim to have done with the microscope in this direction.

The microscope has done, and is doing, a great work in medicine, as well as in the collateral sciences. But I know of no field in which it will be of more practical use than in the treatment of the sterile state. For, where every thing was a short time ago in doubt and confusion, all is now made clear by this wonderful instrument. Even in this day there are many honest cultivators of medical science, who do not believe in the value of the teachings of the microscope.

M. Velpeau died, having no faith whatever in its practical utility. A few years ago I was one of those benighted scoffers who believed it to be merely a scientific toy, with which to while away leisure hours. Fortunately, my ignorance was dispelled, and I now look upon the microscope as an essential to the daily duties of the physician.

With these prefatory remarks, I now beg leave to give you some illustrations of its use in treatment of the sterile state.

In the investigation of any case of sterility, there are three questions that must be settled at the outset, if we expect to treat it understandingly.

1. We must be sure that we have semen with spermatozoa.

2. We must ascertain if the spermatozoa enter the utero-cervical canal.

3. We must determine whether the secretions of this canal are favorable or not to the vitality of the spermatozoa.

For, if the semen does not contain spermatozoa, of course the uterine condition does not call for any treatment whatever. But if it does contain spermatozoa, and if they do not enter the cervical canal, then there is the question of operation or not, to permit their entrance.

On the other hand, if we should find spermatozoa in the cervical canal, then, as a rule, no operation will be needed; and if we should find them there, all or nearly all dead, then it is evident that the secretions of the utero-cervical canal poison them, and therefore the physical condition, giving rise to this abnormal secretion, must be searched out and treated.

The present advanced state of physiological knowledge warrants us in saying that conception is impossible without spermatozoa; and it is impossible if the spermatozoa can not pass into the cavity of the uterus; and, to these acknowledged truisms, I must add another, viz., that it is equally impossible if they die in the cervical canal, or are dead when they reach the uterine cavity. It is, then, self-evident that these three points must be determined—it matters not what other complications may exist. Fortunately, as I have said before, they are all easily and quickly settled by the microscope. Without the microscope, it is impossible to determine either of them. Without the microscope, then, our treatment of the sterile state is simply blind empiricism. With it, our diagnosis becomes absolutely certain, and our treatment, at least rational. What, then, are the first steps in this investigation? How can we begin? Where shall we begin? Now, as it is upon this very point that I have been so stigmatized, I will tell you exactly how I manage this delicate affair.

Given a case of sterility for investigation, the physician examines the state of the uterus and its appendages. His patient may have a frightful dysmenorrhœa, a flexed cervix, a contracted cervical canal, something that would possibly prevent the passage of the semen to the cavity of the uterus. He may feel convinced, in his own mind, that conception can not take place unless some surgical operation be performed—perhaps incision of the cervix uteri. This operation is usually done to permit the passage of the semen into the uterine cavity. But in this, or in any case, what right have we to say that the semen does not or cannot pass into the cavity of the uterus? We must not take it for granted that it does not, simply because the os seems to be small; for we know that cases are recorded where conception occurred when the os barely admitted a small size probe; and we know very well that spermatozoa now and then pass along the Fallopian tubes, which ordinarily admit a bristle. If the semen enter the cervical canal, we may lay it down as a rule, that a dilation of the cervix by incision, or otherwise, is not necessary; but if it do not, it may be necessary. We may perform any rational operation for the relief of suffering, and for the restoration of health; but I insist that we have no right to perform any operation, or to institute any treatment whatever, solely with the view of cure of the sterile condition, till we have first settled the three propositions, above laid down, touching the presence and viability of the spermatozoa. To find out all at once, and, with the least delay and trouble, I usually say to the husband or wife, as it may be, "It is very important, before instituting any treatment, to be sure that the seminal fluid enters the neck of the womb, for without this conception is impossible. We must also ascertain if the uterine secretions kill the semen; if so, a certain treatment will be necessary. If you will, then, send your wife here or come with her any day, five or six hours after coition, it will be easy to settle these points at once." In nineteen cases out of twenty, the wife presents herself the next day. The speculum is introduced (and when I say the speculum, I always mean the one that bears my name), and some vaginal mucus is removed by the syringe, and placed on an object-glass. Then some cervical mucus is drawn out and placed on another object-glass. These two specimens are then examined under the microscope. If we find spermatozoa, well and good; but if we find none, neither in the vaginal nor cervical mucus, our fears are at once aroused. What then is to be done? I simply say that I am not quite satisfied with the examination, and would like to see the wife again, at some future time, under the same circumstances. But suppose we find no spermatozoa on this second examination? Then two questions immediately arise: either, that there are no spermatozoa, or that the semen has all passed off before the case came under observation. Sometimes the semen is all instantly thrown off by the vagina, and then it would not do to pronounce the husband sterile until we are sure of a specimen of his semen for investigation. If I fail to satisfy myself on this point, I then explain the possibility of the semen all passing off in the act of rising and dressing, and show the absolute necessity of making the examination half an hour or so after coition, and before the erect posture is assumed. When the subject is presented in this plain, practical manner, and treated seriously, no man or woman of sense could oppose it; and with me, it has never, in a single instance, been objected to. When I am sent for to make examination, if I find in the vagina a fluid with the characteristic seminal odor, I am satisfied with the micrococoscopic examination. I have never, in but two instances, been compelled to resort to Mr. Curling's plan, of asking the man to squeeze a drop of mucus from the urethra, on a bit of glass, immediately after sexual intercourse. But this is sometimes necessary; it is well to remember it.

If we eventually find that the semen contains no spermatozoa, then all uterine treatment is at an end. But if we are at last satisfied that it contains spermatozoa, then we must determine if these enter the cervix uteri, and if so, do they there find a fluid favorable to their existence alive? And all this can be done only by the microscope.

The question of the entrance of the semen into the cervical canal, and of the effect of its secretion upon the spermatozoa, can be fully and satifactorily ascertained only during a very brief period. We are sure to make a mistake if the microscopic examination be made just before the return of the menses; and why? Because, there is always a certain amount of fullness of the uterus—of engorement, so to speak—which precedes the menstrual flow; and the cervical canal may not admit the semen from mere turgescence of its walls. Besides, at this time, its secretions are almost sure to kill the spermatozoa even if they should happen to enter this canal.

Physiologists are generally agreed that conception takes place during the week following menstruation. Avrard says we have fourteen days of active uterine life and fourteen of uterine hypnotism. He says that conception can occur at any time after menstruation up to the fourteenth day, counting from its commencement. For instance, if menstruation should last for three days, then we would have eleven days for the possibility of conception. But if menstruation should last eight or nine or ten days, then we would have respectively but six or five or four days as the time possible for conception. After this time, the uterus, according to Avrard, lapses into the hypnotic state, when conception is impossible. While I am disposed to accept Avrard's dictum as the rule, I think I have seen exceptions to it, if we can always depend upon testimony seemingly reliable. Be this as it may, I am sure of this fact: if we wish to determine the effects of the cervical mucus upon the spermatozoa, we must make the experiment during the week that follows menstruation. About the fifth or sixth day after the flow is the best moment; for then the uterus is in the most favorable condition. The cervical mucus, which just before menstruation was perhaps thick and opaque, then becomes clear and translucent. If, by examination made at this particular period, we should find spermatozoa in the cervical mucus, we could safely say that it will not be necessary to incise the neck of the uterus. But if the sperm do not enter the canal, then the probabilities are in favor of the necessity of some surgical interference. The semen may enter the cervix in great abundance, and we may find the spermatozoa all dead, even but a few minutes after coition. Then, as said before, we must find out the source of the poisonous secretion, and remedy it; for a vitiated secretion shows some organic condition requiring a special treatment.

When I wish to examine the action of the cervical mucus upon the spermatozoa, I order sexual intercourse in the morning—the dorsal decubitus to be retained for an hour afterward; and I expect a visit from my patient four or five or six hours after coition. Sometimes we find spermatozoa in great abundance in the cervical canal and not one living. (I have occasionally examined the mucus, six, eight and ten minutes after coition, and found all the spermatozoa dead.) Sometimes, we find half of them dead; again, only a third, again, two-thirds. Sometimes, in one portion of the mucus, every spermatozoön is dead; while, in another portion of the same sample with fewer epithelial scales, we may find them alive. Now and then, after treatment for a month or more, I have found the mucus drawn from the lower segment of the cervical canal full of living spermatozoa, and I have supposed that the case was cured; but when I came to examine that drawn from the upper segment of the canal, near the os internum, they were nearly all dead. This was evidently because the mucous membrane lining the lower segment of the cervix, being more easily reached and more thoroughly treated, had assumed a healthier character, and consequently its secretion was restored to a normal condition; while that higher up, and more difficult to reach, had not been so much improved, and hence its secretion was still abnormal—a condition requiring further treatment.

The vaginal mucus, by its natural acidity, kills very quickly every spermatozoön. I do not remember to have found one alive in the vagina, except when the examination was made very soon after coition; when, indeed, the vagina was full of semen but slightly mixed with other secretions. Examined three or four hours after intercourse, the spermatozoa found in that portion of the mucus of the vagina adhering to its walls are all dead. Indeed the normal vaginal secretion seems to be a perfect poison for the superabundant spermatozoa.

When, after a month's treatment, I wish to know whether the case is cured or not—in other words, whether all possible barriers to conception are removed—I order sexual intercourse (just after menstruation) at night, and examine the cervical mucus twelve or fourteen hours afterwards. If the majority of the spermatozoa be alive and active, I have great hope of conception. Before dismissing a case as cured, I think it necessary to examine the mucns thirty-six hours after coition; and, if it is then all right, of course I suspend the treatment, and patiently wait the hoped-for result.

So much care is necessary for the removal of the mucus for the microscopic examination, that I may be pardoned for referring to it again. The patient is placed in the left lateral semi-prone position, as I have elsewhere so minutely described, and my speculum is introduced, and some of the vaginal mucus drawn up with a small glass syringe, previously washed out with warm water. This is deposited on the object.glass; the vagina is then cleared of all secretion, whether vaginal or cervical, the whole of the vagina and the os uteri being thoronghly wiped over with a pledget of cotton. This is for the purpose of guarding against the possibility of mixing vaginal with cervical mucus, which would, of course, spoil the whole experiment. The cervix is then brought forward either by the depressor or a tenaculum, if necessary, which enables us to look directly into the cavity of the cervical canal. The syringe is then again to be thoroughly rinsed in warm water; its nozzle is passed into the gaping canal for half an inch, and the cervical mucus in its lower segment is drawn out. The instrument is emptied, washed out again with warm water and reintroduced up to the os internum, and another portion of mucus is drawn out, provided there is any left after the first effort. Thus we have three specimens of mucus; i. e., one vaginal and two cervical. The cervical secretion should be clear and translucent, and about the consistence of the white of an egg. If it contain any little opaque specks of milky whiteness, it invariably poisons the spermatozoa to a greater or less extent. We sometimes find the cervical mucus perfectly clear, and yet poisonous to the spermatozoa. Here we would naturally expect to find excessive alkalinity of the secretion; but I have not been able to detect it. In these cases it has seemed to me that the spermatozoa were killed—drowned, as it were—by the abundance of the secretion. I do not here allude to cases of uterine catarrh, where the secretion is very thick and albumino-purulent; for, of course, this is a deadly poison to the living principle of the semen. But I allude wholly to such cases as have been changed by treatment to a condition giving rise to a secretion seemingly normal, so far as ordinary ocular examination is concerned. Here the microscope is our unerring guide. The mucus may be clear, and perfectly normal in appearance; but, if it kill the spermatozoa, then there is still some point in the canal of the cervix, or in the cavity of the uterus, that gives out a vitiated secretion; and this must be found out and corrected before the case is wholly cured. When we find living, active spermatozoa high up in the cervical canal thirty-six or forty hours after coition, we can pronounce the case cured, so far as it may be by surgical means, and not till then.

It is time for us to pause, and consider if there is not something more to be done for the sterile condition, than to split up the cervix uteri. I look upon this operation as one of great importance, as one of the most valuable in uterine surgery, but I think we have followed too blindly the example and teachings of its illustrious author, Sir James Y. Simpson. For msself, I am now sure that I have cut open the cervix uteri, perhaps scores of times, when it was both useless and unnecessary; and I know that others have done the same thing. Do not misunderstand me. I speak here solely of the operation with reference to the sterile condition, when it would be wholly useless if the husband happened to be sterile, and certainly unjustifiable unless imperatively called for by considerations of health. Incision of the cervix for dysmenorrhœa is one thing; incision of the cervix for sterility, even if there be dysmenorrhœa, is another, aud it behooves us to draw the line of distinction in every case, and not to take it for granted that every woman is sterile who may have feeble health, and that every man is prolific who may be vigorous and enjoy good health. I am sorry to say that I have had the misfortune to incise the cervix in half a dozen cases of sterility, where I found afterward, to my great mortification, that the husbands are incapable of procreation, because their semen had no spermatozoa, and that, too, since I have known the value of the microscope. In each case the operation was called for to restore health, but was totally useless for the relief of its incidental accompaniment, sterility, and would not probably have been submitted to for considerations of health alone, had it not been for the hope of offspring afterward. I made the mistake of operating on these cases, because the social position, moral character, and appearance of health in the husband, conjoined with the excessive dysmenorrhœa and utter prostration of the wife, led me to operate without the preliminary step of ascertaining whether there were spermatozoa or not. I wish others to profit by my mistakes; and I am less ashamed to tell you of them than I am to own them to myself. However, this can never happen to me again, and should not, after this warning, happen to any of my brethren. I know many men who have no spermatozoa, and cannot therefore become fathers. They are all strong, active men, in the prime of life, and all perform the sexual function with vigor. The very fact of their natural vigor and strong passions had been their ruin, for most of them had contracted urethritis during their early and unmarried life, and had suffered from its unlucky sequence, epididymitis.

Not very long ago (seven or eight years), I had the idea that sterility was essentially a female infirmity; that men were never sterile, except when impotent; and that any man, legally competent for the married state, was physically so for procreation. But the microscope unsettles and settles all such vague notions. It is natural to suppose that a strong, vigorous man is more fitted for procreation than a weak or puny-looking one. Some of the greatest lights of the profession have held such views as this. It was only two or three years before the death of the lamented Trosseau, that he said to me, in speaking of a case we had under consultation, "If our patient only had a man for a husband, all would be right." I subsequently found out that the husband's passions were strong; that his semen was perfect; that it entered the cervix in great abundance; and that the spermatozoa were there poisoned by a vitiated secretion. I mention this to show that we must not judge from appearances, when it is so easy to settle the question by the microscope.

Once I thought that the most common obstacle to conception was a contracted cervical canal, contracted at its outlet, at the os internum, or throughout its entire length. But, if I were now asked, "What is the most frequent obstacle to conception?" I should unhesitatingly say, "An abnormal utero-cervical secretion that poisons or kills the spermatozoa." I can call to mind numbers of cases where, in former years, I incised the cervix, when the operation was satisfactorily done, and yet the sterility persisted. In some of these I have now not the least doubt that the husbands were sterile, and in others I have as little doubt that the cervical mucus was poisonous to the spermatozoa. If I had then possessed the exact knowledge of to-day, how much more satisfactory would it have bave been for me—how much better for my poor patients!

